# 

**Published:** 1898-05

**Authors:** 


					﻿SERIES OF STATE EDITIONS, ft
LEADS VETERINARY JOURNALISM IN AMERICA?
Vol. XIX.
MAY, 1898.
No. 5.
PUBLISHED MONTHLY AT S3 A YEAR. SINGLE COPIES, 30 CENTS.
FOREIGN SUBSCRIPTIONS $3.50 PER YEAR.
THE JOURNAL
OF
Comparative Medicine
AND
VETERINARY ARCHIVES
■
z
o
F
Q
Id
Id
I-
<
I-
U)
£
cc
o
>
3
Id
Z
C=>
30
3*-
-<
m
30
OO
GO
“0
GO
m
GO
3»-
m
ti
GO
”0
m
co
a*
s
so
m
co
-d
—I
-<
—•I
GO
—I
o
"0
GO
m
CO
30
m
GO
3>»
—I
—<
m
30
m
X
—I
3»
EDITED AND PUBLISHED BY
RUSH SHIPPEN HUIDEKOPER, Veterinarian (^.lfoft)A
W. HORACE HOSKINS, D.VxW, I • * 1*- A
H. D. GILL, V.S.juRGEON GENtR/
ALL COMMUNICATIONS AND PAYMENTS SHOULD BE ADDRESSED TO
Dr. W. Horace Hoskins, Managing Editor, 3452 Ludlow St., Philadelphia, Pa.
Copies may be had on order of all news-dealers at home and abroad.
AN ADVERTISING MEDIUM UNEQUALLED IN THE VETERINARY FIELD.
				

